# Developmental and reproductive response of *Brachmia macroscopa* (Lepidoptera: Gelechiidae) to three host plants

**DOI:** 10.1038/s41598-018-27415-z

**Published:** 2018-06-13

**Authors:** Li Ma, Ni Li, Xing Wang, Yan Liu, Ming-Zhu Su, Guo-Hua Huang

**Affiliations:** Hunan Provincial Key Laboratory for Biology and Control of Plant Diseases and Insect Pests, Hunan Agricultural University, Changsha, 410128 P. R. China

## Abstract

The sweet potato leaf folder, *Brachmia macroscopa* Meyrick (Lepidoptera: Gelechiidae), which is a significant pest of plants in the family Convolvulaceae, is rapidly expanding its range in South China and other subtropical regions. Studies were designed to examine the effects of three different host plants (sweet potato, *Ipomoea batatas* (L.) Lam.; water spinach, *I. aquatica* Forsskål; and morning glory, *Pharbitis purpurea* (L.)) on the development and life table parameters of *B. macroscopa* under laboratory conditions. We found that the intrinsic rates of increase of *B. macroscopa* were 0.17 ± 0.004, 0.21 ± 0.005 and 0.16 ± 0.004 on *I. batatas*, *I. aquatica* and *P. purpurea*, respectively. The highest net reproduction rate was 158.06 ± 18.22 per female reared on *I. aquatica*. The larvae had five instars when reared on *I. batatas* and *I. aquatica*, but required six instars on *P. purpurea*. The mean generation lengths of *B. macroscopa* ranged from 24.32 ± 0.18 days when reared on *I. aquatica* to 29.40 ± 0.24 days on *P. purpurea*. The survival of all stage and fecundity curves was intuitively manipulated using the age-stage-structured and two-sex population life table method, to enable comprehensive descriptions of the stage and population trends of *B. macroscopa* on the three Convolvulaceae plants. Our results indicated that *I. batatas* and *I. aquatica* were more suitable host plants than *P. purpurea*.

## Introduction

The relationships between insects and their host plants are considered to be co-evolutionary interactions. In the process of long-term evolution and adaptation to their environment, insects have gradually produced selectivity to their hosts. The host preference may not only influence the growth and development of insects, it may also play an important role in the growth of their population^1^. Feeding on different host plants can also affect the susceptibility of some insect species to certain pesticides^2^. Some studies have shown that the response of insects to the host is based on their demand for external nutrition, while other researchers are of the opinion that the insects are affected by secondary metabolites in the host plants^3,4^. In general, when insects fed on plants that they are adapted to, their growth and survival rates will be optimal and their reproductive capacity will be at a high level, and vice versa^5^. Host plants, in order to discourage insect feeding behavior, are also continually developing an array of defensive strategies, including phenological defenses^6,7^, physical defenses^8^, chemical defenses^9^, etc. The overuse of chemical pesticides to control lepidopteran (and other) pests during the past few decades, has forced targeted insects to evolve in terms of inherent behavioral mechanisms, and altered ecological, genetic and physiological factors, with the end result being the development of resistance to many chemical controls^10^. For example, in using the insecticide fenvalerate in the field to control the diamondback moth, *Plutella xylostella* (L.) (Lepidoptera: Plutellidae), and spraying 10 or more times a year for only two years, has resulted in local populations of the moth that are as much as 12,000 times more resistant to pyrethroid insecticides than other populations^11^.

The sweet potato leaf folder, *Brachmia macroscopa* Meyrick (Lepidoptera: Gelechiidae), is a widely distributed pest in Europe, Russia, Caucasus, Transcaucasian region, West Kazakhstan, central Asia, Korea, Japan, China, and northern India. It mainly damages *Dioscorea esculenta*, *Ipomoea aquatica*, *Calystegia sepium*, *Croomia japonica* and other crops in the Convolvulaceae family. The damage to crops by larvae is significant greater than it is the adult stage. The host plant leaves are damaged by the larvae skeletonizing the surface, leaving only the transparent leaf epidermis; the mesophyll layer of the leaf is completely consumed. The harm caused by *B. macroscopa* larvae seriously affects the ability of the crops to photosynthesize, causing the host plant leaves to wither, and eventually die. The larvae frequently cause extensive damage to their host crops and result in serious economic loss to the growers. Our previous studies have involved determining the complete mitochondrial genomes of this pest, as well as detailed research on the developmental and fecundity responses of the species under different temperatures to identify the most susceptible phase of its development and growth^12,13^. Additionally, based on a two-year field investigation in Changsha, China, we found that local *B. macroscopa* fed mainly on three Convolvulaceae plants, sweet potato vine *Ipomoea batatas* (L.) Lam., Chinese waterspinach *I. aquatica* Forsskål, and Japanese morning glory *Pharbitis purpurea* (L.)^14^. In the present study, we determined different life parameters of *B. macroscopa* by constructing life tables for the moth when reared on each of the three major hosts. The data provides a theoretical basis for devising and implementing efficient as well as environmentally friendly integrated control procedures.

## Results

### Larval stages

*B. macroscopa* was capable of completing its entire life cycle when fed on each of the three host plants tested, although the duration of each stage varied (Table 1). The developmental time of the egg stage was 4 days on *I. aquatica* and *P. purpurea*, which was shorter than eggs laid on *I. batatas* (5 days). The *B. macroscopa* larval developmental durations were significantly different (*P* < 0.0001) when reared on the three plants. When fed on *P. purpurea*, the larvae required six instars to complete development with a developmental period of 14.36 ± 0.14 d, which was the longest total growth period among the three host species. The larval development on *I. batatas* and *I. aquatica* required only five instars, and lasted 12.42 ± 0.11 d and 11.50 ± 011 d, respectively. Significant differences occurred in the developmental duration (*P* < 0.0001) of the 1st and 5th larval stage when reared on the three host plants, but were not found in the 4th instar duration (*P* = 0.536). There was no significant difference between *I. batatas* and *I. aquatica* in the duration of the 2nd instar (*P* = 0.448), or in the 3th instar (*P* = 0.116); nor were there significant differences in the 3th instar stage between *I. aquatica* and *P. purpurea* (*P* = 0.454), although the duration of the 2nd instar when fed on *I. aquatica* was longer than on *P. purpurea*. No significant difference occurred in the 3th instar between *I. batatas* and *I. aquatica* (*P* = 0.116). The 2nd instar larval development duration when fed on *I. aquatica* was longer than on *P. purpurea*. The reverse, however, was true during the 3rd and 4th instars. There were no significant differences between the lengths of the pupal periods after being fed on *I. batatas* and *I. aquatica* (*P* = 0.479). The total durations of the larval developmental periods of *B. macroscopa* fed on the three host plants did show significant differences: the relationship of the lengths was: *P. purpurea* > *I. batatas* > *I. aquatica*, (24.06 ± 0.18 d, 22.50 ± 0.12 d and 20.48 ± 0.10 d, respectively).

**Table 1:** The mean (±SE) duration of immature stages (days) of *Brachmia macroscopa* reared on three hosts under laboratory conditions.

	*Ipomoea batatas*		*Ipomoea aquatica*		*Pharbitis purpurea*		df	*F*	*P*
	n(N)	$\bar{{\boldsymbol{x}}}+{\bf{SE}}$	n(N)	$\bar{{\boldsymbol{x}}}+{\bf{SE}}$	n(N)	$\bar{{\boldsymbol{x}}}+{\bf{SE}}$			
Egg stage	139 (150)	5.00 ± 0.00a	142 (149)	4.00 ± 0.00a	145 (150)	4.00 ± 0.00a	2,423	—	—
1st instar stage	138 (139)	1.85 ± 0.04a	140 (142)	1.15 ± 0.03c	137 (145)	1.33 ± 0.05b	2,412	83.50	<0.0001
2nd instar stage	138 (138)	1.96 ± 0.03a	138 (140)	2.02 ± 0.06a	134 (137)	1.71 ± 0.07b	2,407	9.34	<0.0001
3rd instar stage	135 (138)	2.08 ± 0.02b	137 (138)	2.20 ± 0.06ab	125 (134)	2.26 ± 0.07a	2,394	2.75	0.065
4th instar stage	132 (135)	2.20 ± 0.05a	132 (137)	2.25 ± 0.04a	121 (125)	2.29 ± 0.07a	2,382	0.63	0.536
5th instar stage	129 (132)	4.41 ± 0.08a	125 (132)	3.91 ± 0.06b	121 (121)	2.46 ± 0.07c	2,372	203.71	<0.0001
6th instar stage	—		—		119 (121)	4.43 ± 0.08a	—	—	—
Larval stage	129 (129)	12.46 ± 0.11b	125 (125)	11.50 ± 0.11c	119 (119)	14.36 ± 0.14a	2,370	137.12	<0.0001
Pupal stage	127 (129)	5.04 ± 0.03b	119 (125)	5.10 ± 0.03b	119 (119)	5.68 ± 0.10a	2,362	32.67	<0.0001
Total pre-adult period	127 (127)	22.50 ± 0.12b	119 (119)	20.48 ± 0.10c	119 (119)	24.06 ± 0.18a	2,362	164.04	<0.0001

Note: Data in the table are stated as mean ± SE. Means in the same row marked with different letters are significantly different at the 5% level using the Tukey- Krammer test. n, effective replicate number; $\bar{x}$, mean value; SE, standard error; df, degree of freedom; *F*, value of Levene’s Test; *P*, statistical significance.

### Total duration and longevity of the adult stage

It is evident from Table 2, that when *B. macroscopa* larvae are fed on different host plants, the average lifespans of both male and female adults were significantly different with *P. purpurea* < *I. aquatica* < *I. batatas* (*P* < 0.0001), although there were no significant differences (*P* = 0.101) found in the adult female longevity after feeding on *I. batatas* (31.70 ± 1.27 d) and *I. aquatica* (28.91 ± 1.37 d). Adult females reared on *P. purpurea* had significantly (*P* < 0.05) shorter average life spans (19.90 ± 0.85 d) compared to the other two groups. The lowest larval survival rate, 79.19% occurred when fed on *I. aquatica*, although the rate on *P. purpurea*, 79.33%, was very similar. The highest larval survival rate, 84.67%, occurred in the group fed on *I. batatas*.

**Table 2:** The mean (±SE) duration of adult lifespan, longevity (days) and mortality of immature stages of *Brachmia macroscopa* reared on three hosts under laboratory conditions.

	*Ipomoea batatas*		*Ipomoea aquatica*		*Pharbitis purpurea*		df	*F*	*P*
	n	$\bar{{\boldsymbol{x}}}+{\bf{SE}}$	n	$\bar{{\boldsymbol{x}}}+{\bf{SE}}$	n	$\bar{{\boldsymbol{x}}}+{\bf{SE}}$			
Male longevity	53	34.17 ± 1.46a	61	29.02 ± 1.24b	58	17.48 ± 0.50c	2,169	56.27	<0.0001
Female longevity	74	31.70 ± 1.27a	57	28.91 ± 1.37a	61	19.90 ± 0.85b	2,189	26.67	<0.0001
Male entire lifespan	53	56.26 ± 1.50a	61	49.82 ± 1.19b	58	41.70 ± 0.60c	2,169	39.93	<0.0001
Female entire lifespan	74	54.49 ± 1.27a	57	49.05 ± 1.37b	61	43.80 ± 0.88c	2,189	20.62	<0.0001
Mortality of immature stages	15.33%		20.81%		20.67%		—	—	—

Note: Data in the table are stated as mean ± SE. Means in the same row marked with different letters are significantly different at the 5% level using the Tukey- Krammer test. n, effective replicate number; $\bar{x}$, mean value; SE, standard error; df, degree of freedom; *F*, value of Levene’s Test; *P*, statistical significance.

### Oviposition period and fecundity

Significant differences (*P* < 0.05) were found in *B. macroscopa* adult females reared on the three host plants for the adult pre-oviposition period (APOP; the period of time between the emergence of an adult female and the onset of her first oviposition), the total pre-oviposition period (TPOP; the time interval from birth to the beginning of oviposition), the oviposition period and total fecundity (total number of eggs produced during an individual’s reproductive period) (Table 3). Although the APOP of *B. macroscopa* female adults from *I. batatas* (1.45 ± 0.12 d) and *P. purpurea* (1.39 ± 0.10 d) were similar and not significantly different (*P* = 0.734), the APOP on *I. aquatica* (0.44 ± 0.10 d) was significantly shorter (*P* < 0.05) than the other two groups. The results demonstrated that the fecundity of adult females from *I. aquatica* (427.20 ± 18.08) was significant higher (*P* < 0.05) than it was in the *I. batatas* (298.18 ± 11.42) and *P. purpurea* (299.03 ± 8.86) groups.

**Table 3:** The mean (±SE) duration of oviposition periods (days) and fecundity rates of *Brachmia macroscopa* reared on three hosts under laboratory conditions.

	*Ipomoea batatas*		*Ipomoea aquatica*		*Pharbitis purpurea*		df	*F*	*P*
	n	$\bar{{\boldsymbol{x}}}+{\bf{SE}}$	n	$\bar{{\boldsymbol{x}}}+{\bf{SE}}$	n	$\bar{{\boldsymbol{x}}}+{\bf{SE}}$			
APOP	74 (74)	1.45 ± 0.12a	55 (57)	0.44 ± 0.10b	61 (61)	1.39 ± 0.10a	2,187	23.99	<0.0001
TPOP	74 (74)	24.23 ± 0.18b	55 (57)	20.58 ± 0.15c	61 (61)	25.30 ± 0.23a	2,187	151.32	<0.0001
Oviposition period	74 (74)	18.31 ± 0.51a	55 (57)	12.85 ± 0.46c	61 (61)	14.90 ± 0.65b	2,187	25.93	<0.0001
Total fecundity/female	74 (74)	298.18 ± 11.42b	55 (57)	427.20 ± 18.08a	61 (61)	299.03 ± 8.86b	2,187	30.79	<0.0001

Note: Data in the table are stated as mean ± SE. Means in the same row marked with different letters are significantly different at the 5% level using the Tukey- Krammer test. n, effective replicate number; $\bar{x}$, mean value; SE, standard error; df, degree of freedom; *F*, value of Levene’s Test; *P*, statistical significance.

**Figure 1:**
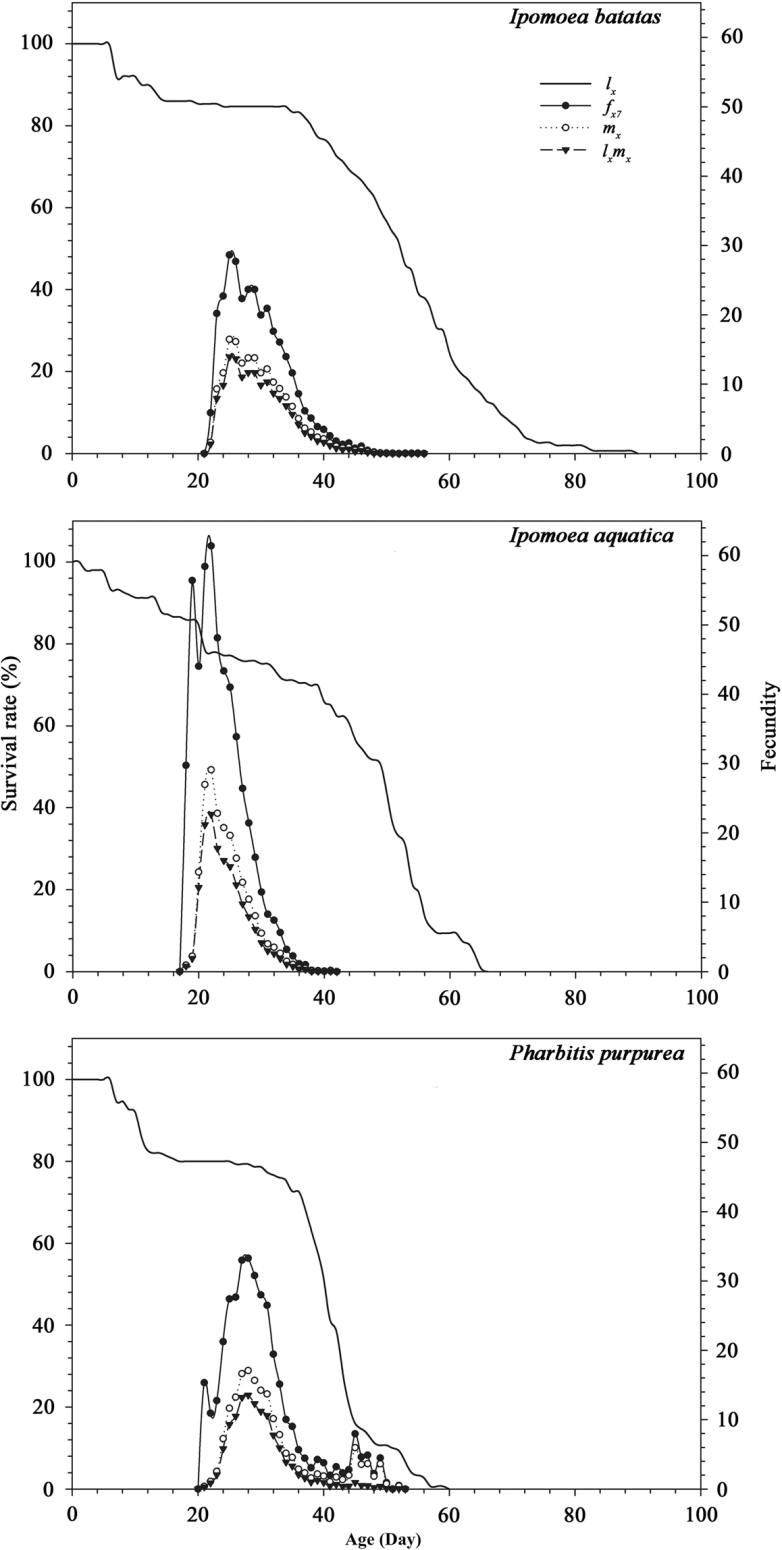
Age-specific survival rate (*l*_*x*_), age-specific fecundity (*m*_*x*_), age-specific morternity (*l*_*x*_*m*_*x*_) and age-stage-specific fecundity (*f*_*x8*_) of *Brachmia macroscopa* reared on three host plants under laboratory conditions.

Since only female adults produce offspring, there is only a single curve representing the female age-stage-specific fecundity (*f*_*x*8_). The peak age-stage-specific fecundity (*f*_*x8*_) on the three different host plants occurred at 25, 22 d, and 28 days with corresponding *fx*_*8*_ values of 27.71, 61.40 and 33.48, respectively (Fig. 1) (single female fecundity per day). The highest and lowest age-specific fecundity values (*m*_*x*_) were found on *I. aquatica* and *I. batatas* separately. The *f*_*x8*_, *m*_*x*_ and *l*_*x*_*m*_*x*_ values for *B. macroscopa* fed on the three plants were in the order: *I. aquatica*, *P. purpurea*, *I. batatas*.

### Survival rate

**Figure 2:**
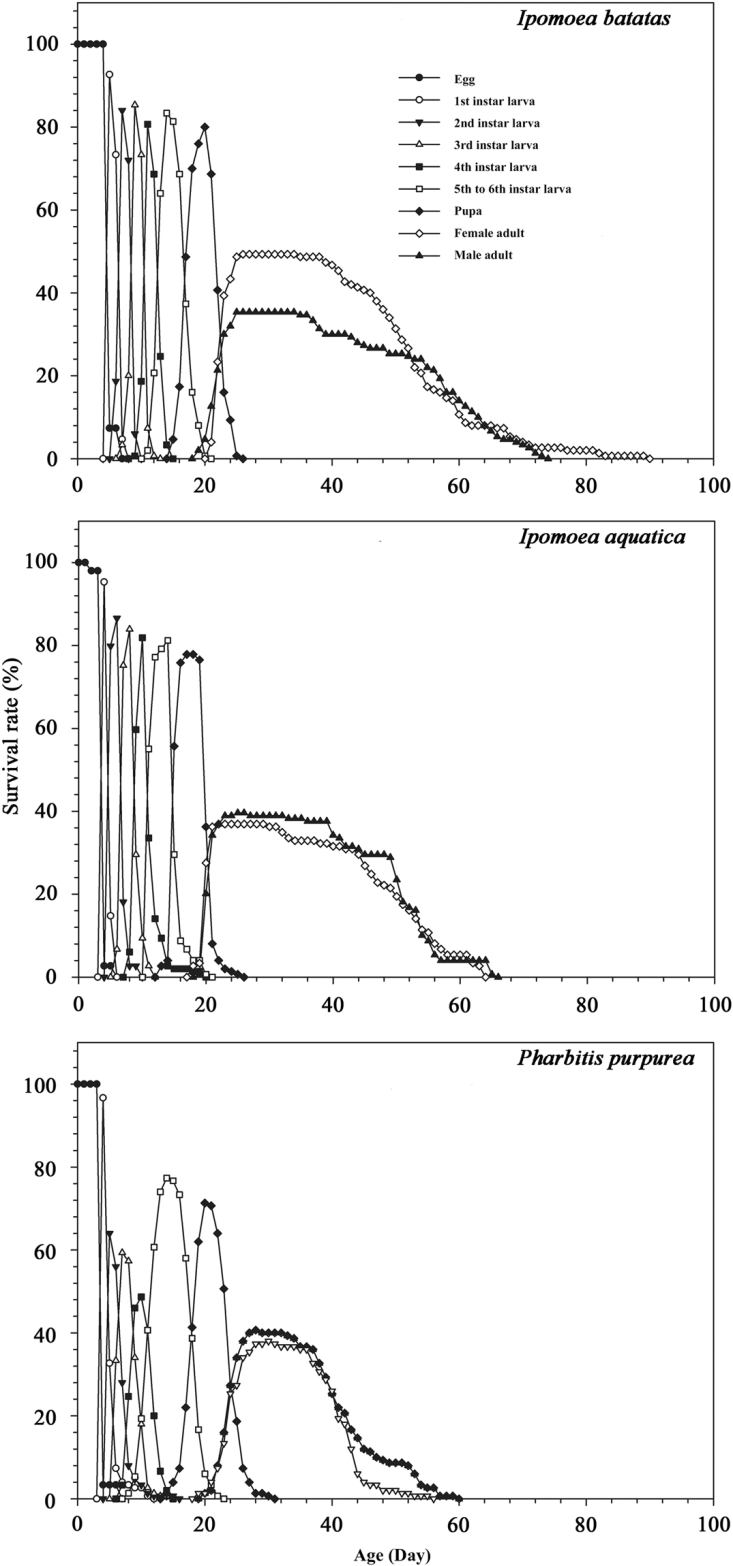
Age-stage-specific survival rate (*S*_*xj*_) of *Brachmia macroscopa* reared on three three host plants under laboratory conditions.

The age-stage-specific survival rates (*S*_*xj*_) of *B. macroscopa* fed on the three different host plants are shown in Fig. 2. It displays the survival rates at various developmental stages (egg, larva, pupa, adult) and the differences in developmental rates of different ages (eggs, 1st instar, 2nd instar, 3rd instar, 4th-6th instar larvae, pupae, and male and female adults), This graphic display demonstrates the overlapping phenomenon of different ages. When *B. macroscopa* larvae fed on *I. batatas*, *I. aquatica* and *P. purpurea*, the probabilities of newly produced eggs successfully developing into adult females were 0.51, 0.62 and 0.59, respectively. Comparable values for the adult males were 0.64, 0.59 and 0.41. The experimental results also showed that the survival rates of different aged *B. macroscopa* fed on *I. batatas* were higher than on *I. aquatica* and *P. purpurea*. The survival rates of the egg, larval and pupal stages of *B. macroscopa* on *I. batatas*, *I. aquatica* and *P. purpurea* were 0.93, 0.93, 0.99, 0.95, 0.89, 0.95, 0.97, 0.83 and 0.99, respectively.

### Life expectancy and reproduction rate contribution value

**Figure 3:**
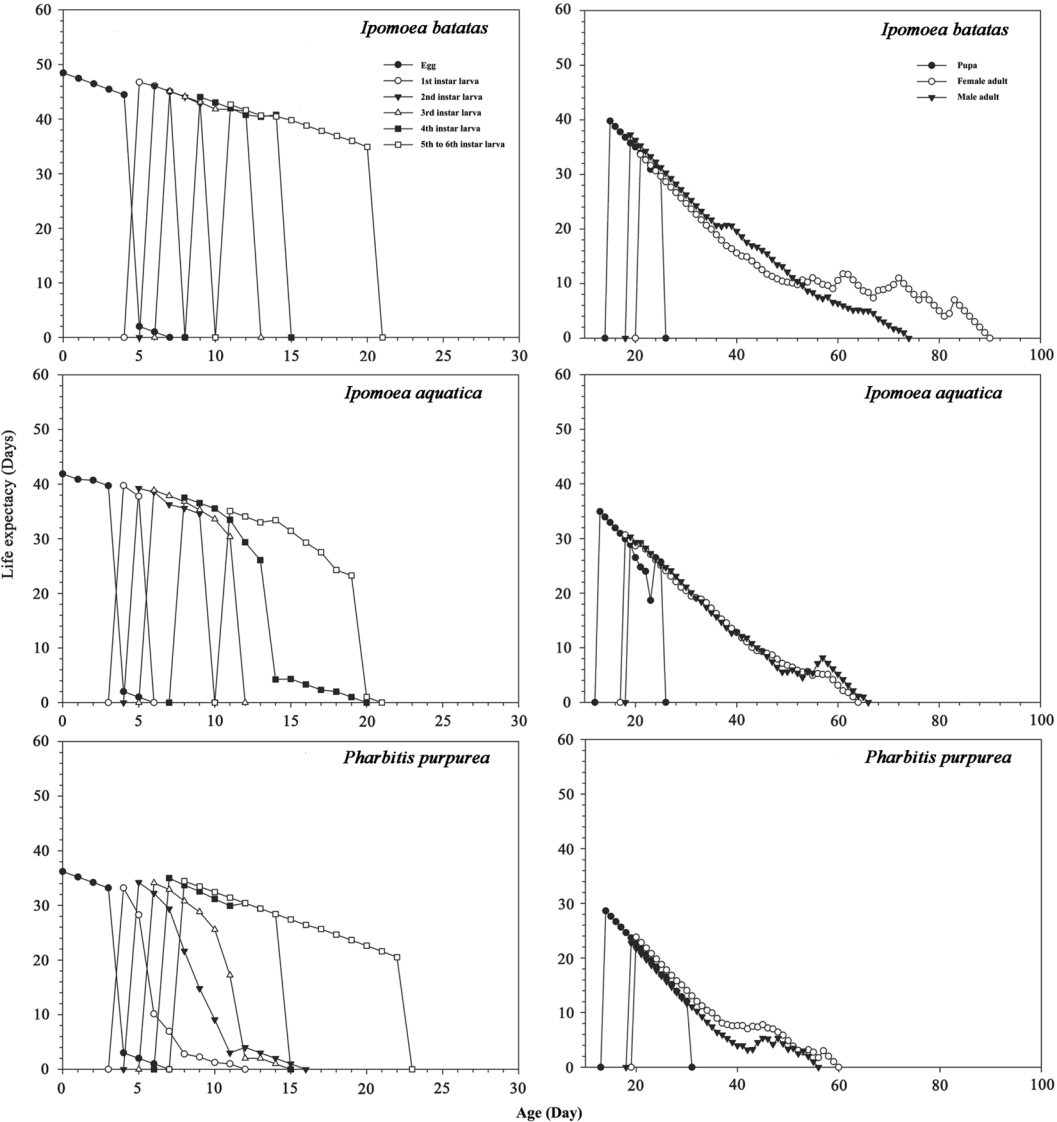
Age-stage-specific life expectancy (*e*_*xj*_) of *Brachmia macroscopa* reared on three three host plants under laboratory conditions.

**Figure 4:**
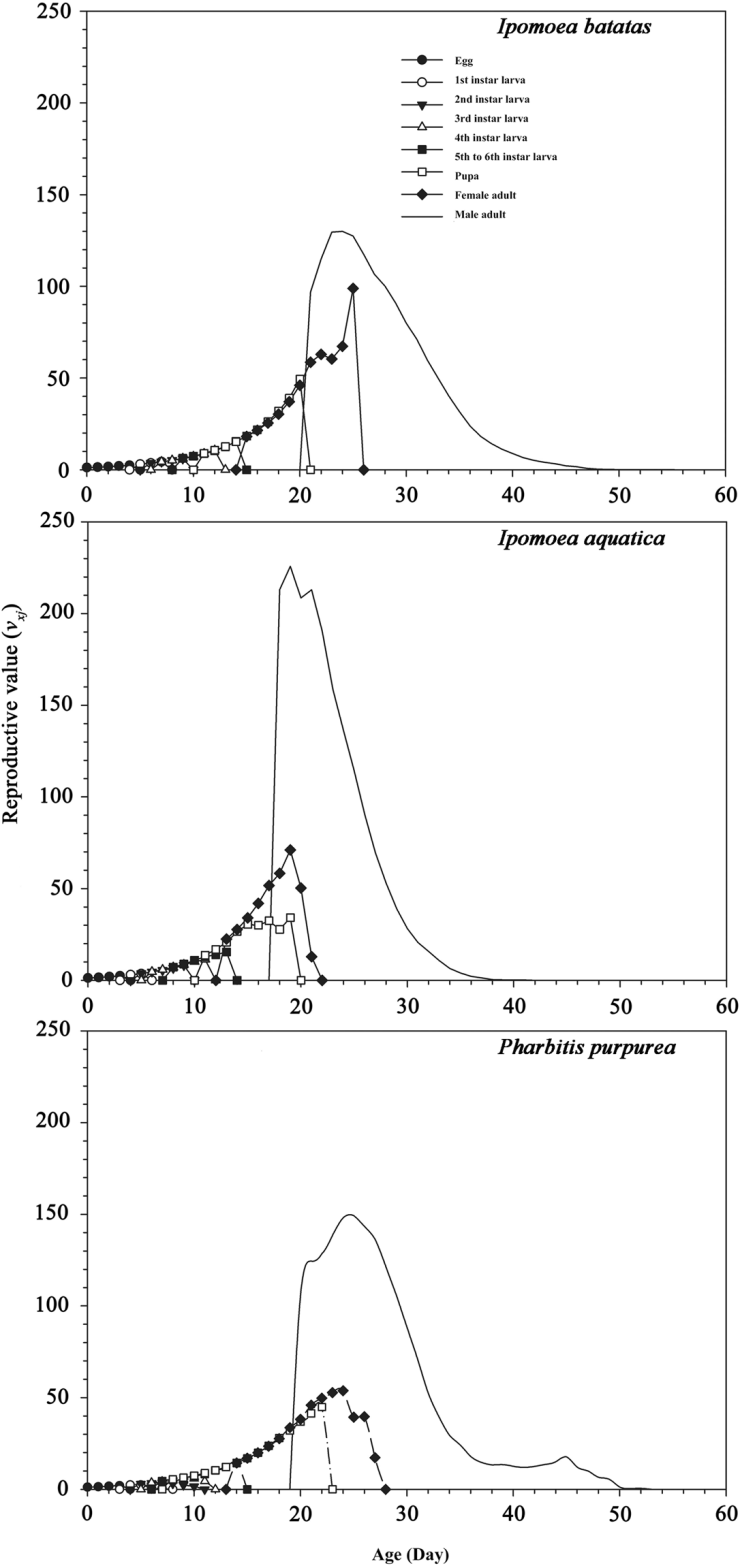
Age-stage-specific life reproductive value (*v*_*xj*_) of *Brachmia macroscopa* reared on three three host plants under laboratory conditions.

The age-stage life expectancy (*e*_*xj*_) is the expected lifespan of different individuals at age *x* and stage *j* when fed on different host plants (Fig. 3). *Brachmia macroscopa* had the longest life expectancy on *I. batatas*, followed by *I. aquatica* and then *P. purpurea*. The results showed that *B. macroscopa* growth on *I. batatas* was relatively slow. The reproductive value (*v*_*xj*_) is the contribution of different individuals at age *x* and stage *j* to future population growth (Fig. 4). The reproductive value reached its highest peak on the 24th day (*V*_24, 7_ = 130.01), 21st day (*V*_21, 7_ = 212.97.01) and 25th day (*V*_25, 7_ = 149.30) when fed on *I. batatas*, *I. aquatica* and *P. purpurea*, respectively.

### Population parameters

The parameters relevant to population development: net reproductive rate (*R*_0_), total reproduction rate (*GRR*), intrinsic rate of increase (*r*), finite rate of increase (*λ*), average mean generation time (*T*), and standard error, that were calculated for *B. macroscopa* populations reared on the three different host plants, are shown in Table 4. There were no significant differences (*P* > 0.05) in the *R*_0_ and *GRR* of *B. macroscopa* fed on the three hosts. The *R*_0_ from high to low value occurred on *I. aquatica*, *I. batatas* and *P. purpurea* (158.06 ± 18.22, 147.59 ± 13.46 and 122.01 ± 12.55), respectively. The highest *GRR* (203.73 ± 21.65) was found in the *I. aquatica* group, followed by *P. purpurea* (177.80 ± 17.87), with the lowest value (175.98 ± 14.80) occurring in the *I. batatas* group. There were no significant differences (*P* > 0.05) in the *r*, finite rate*λ*and *T* of *B. macroscopa* reared on *I. batatas* and *P. purpurea*. The *B. macroscopa* group reared on *I. aquatica*, however, had significantly (*P* > 0.05) higher *r* and *λ* values than the other two groups, although the *T* on *I. aquatica* (24.32 ± 0.18 d) was shorter than it was on *I. batatas* (28.76 ± 0.23 d) and *P. purpurea* (29.40 ± 0.24 d) (*P* < 0.05).

**Table 4:** The mean (±SE) of life table parameters (days) of *Brachmia macroscopa* reared on three hosts under laboratory conditions.

	*Ipomoea batatas*	*Ipomoea aquatica*	*Pharbitis purpurea*
	$\bar{{\boldsymbol{x}}}+{\bf{SE}}$	$\bar{{\boldsymbol{x}}}+{\bf{SE}}$	$\bar{{\boldsymbol{x}}}+{\bf{SE}}$
*R*_*0*_	147.59 ± 13.46a	158.06 ± 18.22a	122.01 ± 12.55a
*GRR*	175.98 ± 14.80a	203.73 ± 21.65a	177.80 ± 17.87a
*r*	0.17 ± 0.004b	0.21 ± 0.005a	0.16 ± 0.004b
*λ*	1.19 ± 0.004b	1.23 ± 0.006a	1.18 ± 0.005b
*T*	28.76 ± 0.23a	24.32 ± 0.18b	29.40 ± 0.24a

Note: Data in the table are stated as mean ± SE. Means in the same row marked with different letters are significantly different at the 5% level using the Tukey- Krammer test.

## Discussion

The development period and fecundity of lepidopterous insects, as well as insect populations in general, are influenced by many factors, including the availability of suitable host plants. The selection of appropriate local host plants plays a vital role in determining whether a natural population will grow or decline. An ideal means of deciphering the factors that enter into this selection is via the use of life tables. Life tables are also useful in providing applied and practical information regarding a given pest’s survival, fecundity and developmental times. Moreover, difference in the individual’s growth and developmental when feeding on different plants can also be determined. Damage caused by *B. macroscopa* to the three widespread hosts selected for this study, *I. batatas*, *I. aquatica* and *P. purpurea*, has been widely recorded in many countries besides China^15^. Because insecticides have traditionally been used to suppress outbreaks of this pest^16^, it is hoped that further understanding the life history of *B. macroscopa* will allow growers to decrease their dependance on pesticides in attempts to control this widespread pest in the future.

In the present study we used the age-stage life table to compare the effects of the above three host plants on the life history parameters of *B. macroscopa*. The age-stage-specific survival rate (*S*_*xj*_) indicates the survival rate at different stages as well as demonstrating the generation overlapping phenomenon. The study of the life table of the geometrid *Problepsis superans* (Butler) on different hosts indicated that at the age of 13 d, the 1st instar larvae coexisted with the 2nd and 3rd instars when *P*. *superans* fed on *Ligustrum lucidum* Aiton, while the insect populations on *L. vicaryi* (Beckett) Rehder and *L. quihoui* (Carr.) coexisted in the 2nd and 3rd instars at 13 d^17^. Alami *et al*. (2014) and Naseri *et al*. (2014) reported the age-stage, two-sex life table of *Chrysodeixis chalcites* (Esper) (Lepidoptera: Noctuidae) and *Helicoverpa armigera* (Hübner) (Lepidoptera: Noctuidae) on different bean cultivars, and described the same phenomenon^18,19^. This shows that the overlapping generations that occur in insect life histories should not be overlooked. While this phenomenon is an integral component of the age-stage life table survival curve, this critical information is lacking in traditional life tables. In the present study, it is evident from Fig. 3 that *B. macroscopa* obviously has this overlapping phenomenon in the development of its immature stages and can exist in multiple stages on any given day.

The egg stage lasted 4 days on *I. batatas* and *I. aquatica*, and 5 days on *P. purpurea*. This was within the range (3–6.35 days) reported by Ma *et al*. when studying the effects of varying temperature on *B. macroscopa*^13^. The length of the egg stage was also close to that observed in several other species of Gelechiidae. A study by Karimi-Pormehr *et al*.^20^ reported that the length of the egg stage of, *Sitotroga cerealella* (Olivier) (Lepidoptera: Gelechiidae) on ten different barley cultivars was from 4.83 to 4.97 days. Rostami *et al*.^21^ reported the egg stage of *Tuta absoluta* (Meyrick) (Lepidoptera: Gelechiidae) lasted from 4.43 to 4.64 days on three tomato cultivars, but other researchers, found the egg stage ranged from 5 to 7 days in the species^22,23^, *B. macroscopa* also has a shorter pupal stage compared to *T. absoluta*. In our study, the *B. macroscopa* pupal stage ranged from 5 to 6 days, which was similar to the length reported under ideal temperatures for *B. macroscopa*^13^. However, according to the recent research on *T. absoluta*, figures ranged from 5 to as much as 10 days, with most moths requiring 8 to 10 days^21–23^. The duration of the total pre-adult stage of *B. macroscopa* lasted 22.50 days on *I. batatas*, 20.48 days on *I. aquatica*, and 24.06 days on *P. purpurea*. These figures are less than those reported for other species in the family Gelechiidae. For example, in *S. cerealella* it was 29.89 to 31.84 days, and *T. absoluta* required 27.09 to 28.10 days^20–23^.

The fecundities of *B. macroscopa* females at 27 °C were 298.18, 427.20 and 299.03 eggs on *I. batatas*, *I. aquatica* and *P. purpurea*, respectively. These figures exceeded those reported for *S. cerealella*, *T. absoluta* and *Cameraria ohridella* Deschka & Dimic (Lepidoptera: Gracillariidae)^20–24^. *S. cerealella* had a maximum fecundity of 80.21 eggs when reared on barley^20^. In *T. absoluta* and *C. ohridella*, the fecundities were consistently less than 200 eggs^21–24^. This high fecundity may explain the multiple outbreaks that are increasingly commonplace in recent years, causing serious damage to a number of crops. Developmental duration and fecundity of the insects were measured to determine whether the herbivorous insects had a good “biological fit” on a particular host plant or not. A short developmental duration and high fecundity may optimize a more suitable environment for the insects^25^. When *B. macroscopa* was reared on *I. aquatica*, the total duration of the egg and larval stages were the shortest, but the survival rates in the larval stage, and the adult fecundity were the highest, suggesting that *I. aquatica* may be the most suitable host for *B. macroscopa*. When evaluating the biological characteristics of an insect population, it is important to not only include the growth and developmental duration, feeding and oviposition abilities^26^, while the *r* and *R*_0_ data, as well as other population dynamics should also be considered^27–29^.

The intrinsic rate (*r*) and net reproductive rate (*R*_0_) of *B. macroscopa* was 0.16 to 0.21 days^−1^ and 122.01 to 158.06 eggs, respectively. These numbers were higher than in some other gelechiid species. Karimi-Pormehr *et al*.^20^ reported that the *r* and *R*_0_ values for *S. cerealella* were 0.077 to 0.115 days^−1^ and 17.17 to 44.09 eggs, respectively depending on the barley cultivar. The *r* and *R*_0_ values for *T. absoluta* were 0.074 to 0.115 days^−1^ and 10.03 to 61.56 eggs, respectively, on different tomato cultivars^21,22^. The *r* of *B. macroscopa* was similar to several noctuid species, *e.g*., in *Helicoverpa armigera* it was 0.115 to 0.142 days^−1^ and 0.171 to 0.264 days^−1^ in *Spodoptera exigua* (Hübner)^19,30^, but the *R*_0_ values were less than those in *H. armigera* (177.3 to 270.1 eggs) and *S. exigua* (126.4 to 377.1 eggs)^17,30^. The *r* value of *B. macroscopa* was smaller than that reported for *P. xylostella* which exceeded 0.2 days^−1^ ^31,32^. The *R*_0_ of *P. xylostella* differed significantly on various host plants ranging from 30.6 to 183.8 eggs^31,32^. The *R*_0_ of *B. macroscopa*, under an ideal temperature of 27 °C, was reported as 147.60 eggs by Ma *et al*.^13^. Our results approximated theirs (122.01 to 158.06 eggs). The *B. macroscopa* population appeared to be especially stable, since its host selectivity was not especially strong.

The primary reason for animals (including insect herbivores) to eat is to acquire a mix of nutrients in order to complete the processes of growth, development, and reproduction^33^. On the contrary, different host plants can also affect insects’ growth and reproduction, which can potentially lead to a change of their life history^34,35^. These changes are often vital to the insect’s population dynamics in the wild. The nutrition contained in a host plant determines whether it suitable for an insect population or not. Of prime importance, the quality and quantity of the plants can affect the food digestibility and fecundity of a pest; secondarily, a high fecundity and short TPOP will directly lead to a high intrinsic rate^19^. When *B. macroscopa* larvae fed on *I. aquatica*, the total fecundity was higher than in the other two hosts with 427.60 eggs, the TPOP was the shortest with 20.58 days and the intrinsic rate (*r*) was also the highest, as were the *R*_0_, *GRR* and *λ* values. These results would suggest that *I. aquatica* is the most suitable host for *B. macroscopa*. The *r* and *λ* values of *B. macroscopa* were the second highest on *I. batatas*. Insects on *I. batatas* had the shortest pupal period as well as the longest adult duration. These results indicated that *B. macroscopa* was able to develop adequately on *I. batatas*, did not do well on *P. purpurea*. Finally, *B. macroscopa* apparently has considerably stronger host adaptability on *Ipomoea* than it does on *Pharbitis* plants.

## Materials and Methods

### Tested plants

Based on preliminary field investigations conducted in Changsha, Hunan Province, China, we screened *I. batatas*, *I. aquatica*, and *P. purpurea* to use as hosts for our research. All plants used in the research were grown in pesticide free environments.

### Insect

The colony of *B. macroscopa* used in this study was collected from an experimental farm in Hunan Agricultural University, Changsha, Hunan Province, China; N28°110′, E113°40′. The larvae were cultured separately on fresh leaves of the three host species (*I. batatas*, *I. aquatica*, and *P. purpurea*) in the laboratory. Newly emerged females and males were individually paired in an insect rearing cage containing young plants of each host to encourage mating and to act as an oviposition substrate. A mesh net and a cotton ball soaked with a 20% honey solution were placed in the rearing cages for ventilation and nutrition, respectively. The insects used in the experiments had been laboratory bred for at least two generations on each host species. All tested insects were maintained in an artificial climate chamber at 27 ± 1 °C, 75 ± 20% RH and 10 L: 14D, and reared on one of the three host plants mentioned above.

### Experiments

One hundred and fifty healthy, well developed, recently laid eggs that were oviposited during the spawning peak, were collected and placed in an artificial climate chamber set at 27 ± 1 °C, 75 ± 20% RH, and a photoperiod of 10:14 (L: D) h. Hatching was observed and recorded daily. The newly hatched larvae were transferred using a brush into petri dishes (15 cm in diameter, 1.5 cm in height) and provided with fresh leaves from *I. batatas*, *I. aquatica* or *P. purpurea* plants. The petioles of these leaves were inserted into water-soaked cotton to maintain freshness. The larval mortality was recorded and the leaves replaced daily. Newly pupated individuals were transferred into separate glass tubes (2 cm in diameter, 10 cm in height) with a water-soaked cotton ball to retain moisture and covered with gauze. The pupae were observed daily until emergence as adults. Detailed records were kept on the durations and survival of each larval instar, pupal stage and adult longevity. Upon eclosion, females and males emerging within a 24 h period from each of the host plants were paired and maintained in a plastic oviposition chamber (13 cm in diameter, 17 cm in height). A small host plant, planted in a disposable cup, was added to each container as an oviposition substrate. A cotton ball soaked with a 20% honey solution was placed in the cup to serve as a food source for adults and was replaced daily. The numbers of newly produced eggs were totaled every 24 h until all the adults had died.

### Life table parameters

The age-stage, two-sex life table method was used to analyze the raw data. The age-stage-specific survival rates (*s*_*xj*_) were determined for each of the three treatments. These are the survival rates at age *x* (days) and stage *j* (where the first through eighth stages represent the egg stage (1), the 1st through 4th larval instars (2–5), the 5th (or combined 5th + 6th - when present) instars (6), the pupal stage (7) and the adult stage (8), respectively)^36^. The age-stage specific fecundity (*f*_*xj*_) is defined as the quantity of eggs produced by each individual per day at age *x* (days) and stage *j*; the age-specific survival rate (*l*_*x*_) is the probability that a newly produced egg will survive to age *x*; the age-specific fecundity (*m*_*x*_) is the mean number of female eggs laid per female adult at age *x*. The population parameters were calculated according to the procedure developed by previous researchers^36^. The intrinsic rate of increase (*r*) was calculated using the iterative bisection method where ${\sum }_{x=0}^{\infty }{e}^{-{r}_{m}(x+1)}{l}_{x}{m}_{x}=1$^37^. The net reproductive rate (*R*_0_) was estimated as ${\sum }_{x=0}^{\infty }{l}_{x}{m}_{x}$. The life parameters *GRR* (total reproductive rate), *λ* (finite rate of increase) and *T* (mean generation time, which is the duration that a population needs to increase to *R*_0_-fold its size before reaching the stable age-stage) were calculated as GRR = $\sum {m}_{x},\,\lambda ={e}^{{r}_{m}}$ and *T* = (ln*R*_0_)/*r*_*m*_. Finally, the means, standard errors and variances of the population parameters were bootstrapped using the computer program TWOSEX-MSChart^38^. The graphs were created using Sigma plot 12.5.

### Data analysis

The raw life-history data of *B. macroscopa* reared on each host species were entered separately into Microsoft Excel 2015, and the data analyzed by one-way ANOVA using SPSS 22.0. The correlation coefficient differences were compared using the Tukey method (*P* < 0.05).
