# Author Correction: Developmental and reproductive response of *Brachmia macroscopa* (Lepidoptera: Gelechiidae) to three host plants

**DOI:** 10.1038/s41598-020-78822-0

**Published:** 2020-12-16

**Authors:** Li Ma, Ni Li, Xing Wang, Yan Liu, Ming-Zhu Su, Guo-Hua Huang

**Affiliations:** Hunan Provincial Key Laboratory for Biology and Control of Plant Diseases and Insect Pests, Hunan Agricultural University, Changsha, 410128 P.R. China

Correction to: *Scientific Reports* 10.1038/s41598-018-27415-z, published online 13 June 2018.

The Article contains errors in the Materials and Methods section under the subheading ‘Life table parameters’.

“The age-stage, two-sex life table method was used to analyze the raw data.”

should read:

“The age-stage, two-sex life table method^1,2^ was used to analyze the raw data.”

In addition, the sentence,

“Finally, the means, standard errors and variances of the population parameters were bootstrapped using the computer program TWOSEX-MSChart^38^.”

should read:

“The age-stage life expectancy (*e*_*xj*_) was calculated according to Chi and Su^4^, while the reproductive value (*v*_*xj*_) was calculated according to Tuan et al.^5^ and Tuan et al.^6^. Finally, the variances and standard errors of the population parameters were estimated by using bootstrap technique embedded in the computer program TWOSEX-MSChart^38,3^.”
